# Supplementary material for: AKBA Promotes Axonal Regeneration via RhoA/Rictor to Repair Damaged Sciatic Nerve
Source: Int J Mol Sci. 2022 Dec 14;23(24):15903. doi: 10.3390/ijms232415903 (PMC9783960; doi:10.3390/ijms232415903)
Supplement: Supplementary file 1 [file ijms-23-15903-s001.zip › ijms-2017511-supplementary.pdf]

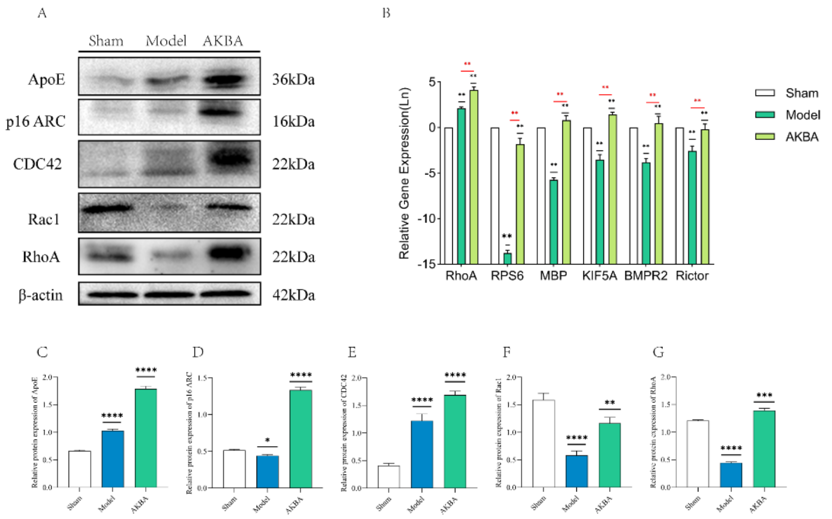

Figure S1. RT-qPCR and Western Blot to verify proteomics. A: Immunoblot of ApoE, p16 ARC, CDC42, Rac1 and RhoA. B: RT-qPCR of RhoA, RPS6, MBP, KIF5A, BMPR2 and Rictor.

C: Quantification of ApoE immunoblots. D: Quantification of p16 ARC immunoblots. E: Quantification of CDC42 immunoblots.

F: Quantification of immunoblot of Rac1. G: Quantification of immunoblot of RhoA.

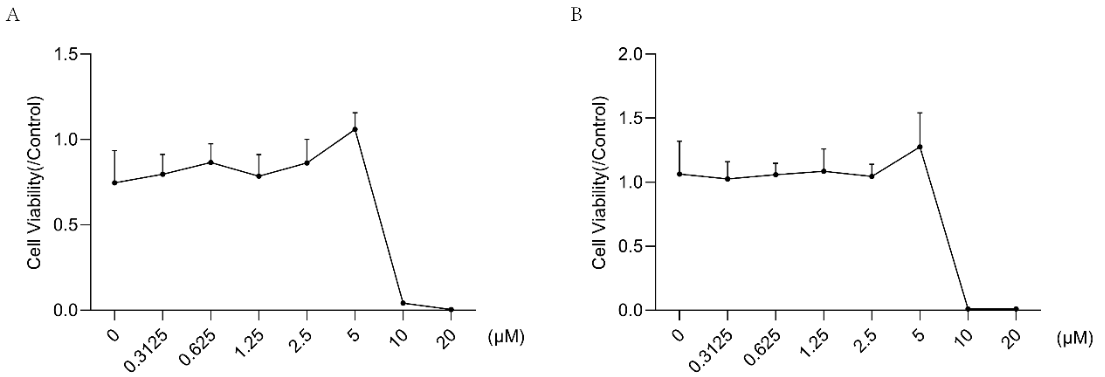

Figure S2. Proliferative and toxic effects of different concentrations of AKBA on PC12 cells. A: Cell viability of PC12 cells after 12 h of different concentrations of AKBA. B: Cell viability of PC12 cells after 24 h of different concentrations of AKBA.

B: cell viability of PC12 cells after 24 h of AKBA.

Table S1 Primer sequence list.

Target gene	Primer sequence
Rho A	Forward：5’-AGGATTGGCGCTTTTGGGTA-3’
	Reverse：3’-ATGAGGCACCCCGACTTTTT-5’
RPS6	Forward：5’-GCGCCTGCTTTTGAGTAAGG-3’
	Reverse：3’-CAGGTTGGCATCCACAATGC-5’
MBP	Forward：5’-CGCAGAGGACCCAAGATGAA-3’
	Reverse：3’-AAATCTGCTGAGGGACAGGC-5’
KIF5A	Forward: 5’-CACGACTCAGGAGCAGGTTT-3’
	Reverse：3’-TGGTTGAAGATGTCTCGGGC-5’
BMPR2	Forward: 5’-CAAGAACGGCTGTGTGCATT-3’
	Reverse: 3’-CAGACCATAACATGTGCTCCCT-5’
Rictor	Forward: 5’-TGCACCCATCCTTGTCTCAC-3’
	Reverse: 3’-CTGATTCCCCCGACATGCTT-5’
GAPDH	Forward: 5’-GCATCTTCTTGTGCAGTGCC-3’
	Reverse: 3’-GATGGTGATGGGTTTCCCGT-5’
